# Increased Hazard Ratio of a Second ACL Injury After Return to Sport for Each Positive Hypermobility Test on the Beighton Score: A Registry Study

**DOI:** 10.1186/s40798-026-01054-5

**Published:** 2026-06-23

**Authors:** Jakob Lindskog, Bálint Zsidai, Axel Sundberg, Johan Högberg, Rebecca Hamrin Senorski, Behnam Liaghat, Kristian Samuelsson, Roland Thomeé, Ramana Piussi, Eric Hamrin Senorski

**Affiliations:** 1 Unit of Physiotherapy, Department of Health and Rehabilitation, Institute of Neuroscience and Physiology, Sahlgrenska Academy, University of Gothenburg, Box 455, 405 30 Gothenburg, Sweden; 2 Sahlgrenska Sports Medicine Center, Sahlgrenska Academy, Gothenburg, Sweden; 3 Department of Orthopedics, Skåne University Hospital, Malmö/Lund, Sweden; 4 Center for Muscle and Joint Health, Department of Sports Science and Clinical Biomechanics, University of Southern Denmark, Odense, Denmark; 5 Department of Orthopaedics, Institute of Clinical Sciences, Sahlgrenska Academy, University of Gothenburg, Gothenburg, Sweden; 6 Department of Orthopaedics, Sahlgrenska University Hospital, Mölndal, Sweden

**Keywords:** Anterior cruciate ligament reconstruction, RTS, Postoperative complications, Graft rupture, Contralateral ACL injury, Joint instability

## Abstract

**Background:**

Generalized joint hypermobility (GJH) has been associated with increased risk of second anterior cruciate ligament (ACL) injury. The clinical diagnosis of GJH relies on a binary threshold of positive joint hypermobility tests, based on age and patient sex, which may overlook the degree of hypermobility.

**Objectives:**

To analyze the association between the number of positive joint hypermobility tests on the Beighton Score and the hazard of second anterior cruciate ligament (ACL) injury in patients who return to sport (RTS) after primary ACL reconstruction, which included secondary, stratified analyses of graft rupture and contralateral ACL injury.

**Design:**

Registry study.

**Methods:**

Data were extracted in January 2026 from an ACL-rehabilitation-specific registry, Project ACL. Included patients were 15–50 years who underwent primary ACL reconstruction with hamstring tendon or bone–patellar tendon–bone autograft, had a documented Beighton Score, participated in knee-strenuous sports before injury, had RTS, reported second ACL injury or had ≥ 1 year follow-up without second ACL injury after RTS. Multivariable Cox proportional hazard regression was used to estimate the cause-specific hazard ratio (HR) of second ACL injury (measured from RTS) based on the Beighton Score, adjusted for age, return to pre-injury physical activity level or higher, graft choice, knee hyperextension (≥ 10° knee extension), and patient sex, accounting for competing risks.

**Results:**

The analysis included 935 patients (mean age 23.7 ± 7.8 years, 51.4% female), with median Beighton Score of 2 (interquartile range: 4). The median follow-up time was 54.4 months. The cumulative incidence of second ACL injury after RTS was 13.1%. Specifically, the cumulative incidence for graft rupture was 7.4% and 5.7% for contralateral ACL injury. For the primary outcome, each additional positive joint hypermobility test on the Beighton Score was associated with a significantly increased hazard of second ACL injury (adjusted HR: 1.10 (95% CI 1.003–1.22, *p* = 0.044). For the secondary outcomes, each additional test was associated with an increased hazard of graft rupture (adjusted HR: 1.15, 95% CI 1.01–1.30, *p* = 0.031), whereas no statistically significant association was observed for contralateral ACL injury.

**Conclusions:**

The HR of second ACL injury (graft rupture or contralateral ACL injury) after RTS in patients who had undergone primary ACL reconstruction increased by 10%, and by 15% for isolated graft rupture, for each positive joint hypermobility test on the Beighton Score, while no association was observed for contralateral ACL injury.

## Key Points

- There was an increased hazard ratio of second ACL injury for each positive test on the Beighton Score after return to sport after primary ACL reconstruction.
- Clinicians should consider the number of hypermobile joints in conjunction with presence of generalized joint hypermobility when assessing risk for second ACL injury.

## Introduction

Patients who sustain an anterior cruciate ligament (ACL) injury typically participate in cutting or pivoting sports [1]. For patients who aim to return to sport (RTS), the current standard of care for the ACL injury consist of ACL reconstruction [2], typically performed with an autograft [1]. Despite ACL reconstruction, up to 20% of all patients, and 23% of patients younger than 25 years, sustain a second ACL injury after RTS [3]. A previous systematic review [4] reported similar rates of graft rupture (11%) and contralateral ACL injury (12%) after ACL reconstruction, although the use of hamstring tendon autograft was associated with increased likelihood of graft rupture compared to the bone–patellar tendon–bone autograft, while patients < 18 years had increased risk for contralateral ACL injury.

An additional factor proposed to increase risk of second ACL injury is generalized joint hypermobility (GJH) [5]. The GJH phenotype is a congenital condition characterized by an altered structural integrity of collagen fibrils, which consequently leads to joint hyperextensibility compared to individuals without GJH [6]. The current consensus for assessment of GJH recommends the Beighton Score, which relies on 9 joint mobility tests, where the number of positive tests confirms GJH based on age and patient sex-dependent cutoff thresholds [7, 8]. Currently accepted thresholds for GJH based on the Beighton Score include ≥ 5/9 points for pre-pubertal males, and ≥ 4/9 points for post-pubertal males [8, 9]. Females with pre- and post-puberal age criteria require an additional point to be assigned the GJH phenotype (≥ 6/9 and ≥ 5/9, respectively) [8]. However, cautious interpretation of the Beighton Score is warranted, as joint mobility can be confounded by various factors, such as traumatically acquired joint hyper- or hypomobility [10, 11]. The use of predetermined binary cutoff values to classify GJH could make accurate classification difficult, which could potentially lead to over- or underestimation of GJH prevalence, and incorrect second ACL injury risk stratification. Given that joint hypermobility exists on a spectrum [12], the associated risk of a second ACL injury is also likely to vary along a continuum. Accordingly, the total number of hypermobile joints may offer valuable insight into individual risk profiles. Despite this, the association between the number of positive joint hypermobility tests on the Beighton Score and the occurrence of second ACL injury has been scarcely studied, but may provide a more informative alternative for second ACL injury risk assessment than relying on predetermined binary cutoffs.

The purpose of this study was to analyze the association between the number of positive joint hypermobility tests on the Beighton Score and the hazard of second ACL injury in patients who RTS after primary ACL reconstruction, which included secondary, stratified analyses of graft rupture and contralateral ACL injury. We hypothesized that there would be an increased HR based on the increased number of positive hypermobile joints on the Beighton Score.

## Methods

### Study Design

The present study was a registry-based retrospective cohort study performed in accordance with the REporting of studies Conducted using Observational Routinely-collected health Data (RECORD) statement [13]. Ethical approval has been obtained from the Swedish Ethical Review Authority (registration numbers: 2020-02501, 2024-08724-01), and the Regional Ethical Review Board in Gothenburg, Sweden (registration numbers: 265-13, T023-17).

### Setting

Data were extracted from a rehabilitation specific registry (Project ACL), previously described in detail [14, 15]. Project ACL was established in 2014 in Gothenburg, Sweden, with the aim to improve care for patients after ACL injury. Patients registered in Project ACL are invited to participate in supervised muscle function tests and answer web-based questionnaires at predefined follow-ups at 10 weeks, 4, 8, 12, 18, 24 months, 5 years and then every fifth year after ACL injury or reconstruction. Patients may register to Project ACL at any time after ACL injury or reconstruction. Upon registration in Project ACL, patients are required to provide demographic information, such as patient sex, date of birth, height, and weight. Joint hypermobility assessment is performed at the initial muscle function test and is conducted by a trained test supervisor. The Beighton Score was added to Project ACL’s standardized assessment protocol in 2019.

### Patients

Patients registered in Project ACL were checked for eligibility for inclusion by application of the following criteria: underwent primary ACL reconstruction performed with hamstring tendon- or bone–patellar tendon–bone autograft, were aged between 15 and 50 years at the time of primary ACL reconstruction, had registered Beighton Score with specific information on the presence of knee hyperextension, pre-injury participation in knee-strenuous sports, defined as ≥ 6 on the Tegner Activity Scale [16] (Tegner), RTS after primary ACL reconstruction, i.e., ≥ 6 on the Tegner, and sustained a second ACL injury (defined as graft rupture or contralateral ACL injury), or had ≥ 1 year follow-up after RTS without a second ACL injury.

Patients with ≥ 3 ACL injuries were excluded. The rationale for the chosen inclusion criteria was to study a broad spectrum of patients who were active in sports prior to primary ACL injury, and to allow adequate exposure time to sustain a second ACL injury after RTS. Only patients with the hamstring tendon and bone–patellar tendon–bone autografts were included due to low numbers of the other graft types.

### Outcomes

The primary outcome was the occurrence of second ACL injury (either graft rupture or contralateral ACL injury). Secondary outcomes were graft rupture and contralateral ACL injury analyzed independently. The primary exposure of interest was the number of positive hypermobility tests on the Beighton Score. Patients were followed until the occurrence of second ACL injury or the date of data extraction (January 2026).

### Variables

#### Occurrence of Second ACL Injury

A second ACL injury (graft rupture or a contralateral ACL injury) can be reported by the responsible clinician or by the patient, after confirmation through clinical assessment or imaging. The occurrence of a second ACL injury is documented in the patient’s profile within the Project ACL database.

### Tests of Hypermobility

The Beighton Score assesses joint hypermobility on a 9-point scale [7], and has been reported with a substantial to excellent inter- and intrarater reliability (intraclass correlation coefficient: 0.72–0.91, and 0.89–0.98, respectively) for raters, regardless of expertise, evaluated in patients between 4 and 71 years old in various settings [17]. The Beighton Score comprises 9 tests, where each positive test yields one point on the score, up to a maximum of 9 points. Eight of the tests are unilateral, and one is bilateral. The eight unilateral tests are performed bilaterally and consist of dorsiflexion of the metacarpal joints of the fifth finger beyond 90°, apposition of the thumbs to the flexor aspects of the forearms, hyperextension of the elbows and knees beyond 10°, and the one bilateral test is forward flexion of the trunk, with the knees straight, so that the palms of the hands can rest easily on the floor [7]. The Beighton Score is currently the most frequently used assessment tool for GJH [9].

In Project ACL, the unilateral tests are passively performed by a test supervisor. The trunk flexion test is performed actively by the patient. The assessment can be performed both prior and after ACL reconstruction, dependent on when the patients perform the first muscle function tests. To account for the potential altered joint mobility due to the ACL injury or reconstruction, the use of an injury allowance point is applied [18]. Application of an injury allowance point means to assign the result of the non-involved limb to the involved limb if the involved limb has altered joint mobility. In Project ACL the Beighton Score assessment is entered into the database as a total score, and a binary categorical variable (yes/no) is reported for the prevalence of knee hyperextension (≥ 10° knee extension).

### Measurement of Knee-Strenuous Activity and RTS

The Tegner is a self-reported questionnaire used to quantify the level of knee-strenuous activity performed before and after an injury [16]. The intraclass correlation coefficient for test–retest reliability for the Tegner was reported to be 0.8 [19]. The original Tegner ranges from 0 to 10, where 10 represents highest knee demanding activity, such as professional football [16]. Project ACL utilizes a modified version of the Tegner that ranges between 1 to 10. Tegner level 6 was the cutoff used to determine RTS in the present study, as Tegner level 6 is the first level determined as knee-strenuous and only includes sport activities, such as recreational ice-hockey, tennis, alpine skiing, or volleyball, or competitive bandy, orienteering, or snowboard [16], and has previously been used to classify knee-strenuous sports participation [20].

### Data Collection

Data were extracted from Project ACL for analysis on January 5, 2026. Data consisted of categorical data of patient sex, choice of autograft for primary ACL reconstruction, Beighton Score, presence of knee hyperextension, as well as occurrence and time of second ACL injury. Furthermore, continuous demographic variables which include age, height, weight, body mass index, time between primary ACL injury and reconstruction, time between ACL reconstruction and RTS, follow-up time after RTS, and level of sport pre-injury and at RTS were extracted.

### Statistical Analysis

Continuous data were presented as mean with standard deviation (SD) for normally distributed data and median with interquartile range (IQR) for non-normally distributed data. Normality distribution check included visual inspection of histograms and Q–Q plots. Categorical data were presented as numbers with percentage. Follow-up time after RTS was analyzed with the Kruskal–Wallis test between patients with different total Beighton Scores. Cause-specific hazard ratios (HRs) of second ACL injury were determined using multivariable Cox proportional hazard regression models and was presented with a 95% confidence interval (CI), accounting for competing risks. The time component of the multivariable Cox proportional hazard regression models was the time from RTS to second ACL injury or time of data extraction. Hazard ratios were interpreted as the relative rate of sustaining the event over time between groups. For the primary outcome, the dependent variable was the dichotomized occurrence of second ACL injury after RTS (yes/no), and patients who did not sustain a second ACL injury were censored at time of data extraction. For the primary outcome, Kaplan–Meier curves were used to illustrate event-free survival over time. For the secondary outcomes, the dependent variable was the dichotomized occurrence of graft rupture or contralateral ACL injury were analyzed as competing events. Cause-specific HRs were estimated using multivariable Cox proportional hazard regression models, with patients censored at the time of competing event (i.e., graft rupture in the contralateral ACL injury analysis, and contralateral ACL injury in the graft rupture analysis). For the secondary outcomes, cumulative incidence functions were used to illustrate the probability of graft rupture and contralateral ACL injury over time, accounting for competing risks. For the unadjusted and adjusted analysis, the independent variable was the number of positive joint hypermobility tests assessed with the Beighton Score. For the adjusted analysis, age at primary ACL reconstruction, return to pre-injury Tegner or higher (yes/no), autograft choice (hamstring tendon or bone–patellar tendon–bone), presence of knee hyperextension, and patient sex were added as confounders to the model based on their relevance in current literature [21–30]. Given that knee hyperextension is included as a component of the Beighton Score, the models were refitted without this variable, with result estimates were materially unchanged, which indicates that the inclusion did not meaningfully affect the observed associations. For all outcomes, the adjusted models were checked for performance with the Grambsch and Therneau’s test for proportional hazards assumption, Harrell’s C-statistic for model discrimination, likelihood ratio test for model fit, and adding a quadratic term to check the assumption for log-linearity for the Beighton Score. For model discrimination, Harrell’s C-statistic was used and interpreted as follows: < 0.60 poor discrimination; ≥ 0.6 to < 0.75 possibly helpful discrimination; and ≥ 0.75 useful discrimination [31]. For model fit, the likelihood ratio test assesses whether adding covariates to the model improves the ability to explain variation in hazard compared to a model without covariates, where a good fit is considered if *p* < 0.05. The log-linearity assumption was considered fulfilled if *p* > 0.05. Alpha was set to *p* < 0.05. Analyses were performed using complete case analysis, including only patients with available data for the outcome and all covariates. Statistical analyses were performed with the Statistical Product and Service Solutions (IBM Corp. Released 2017. IBM SPSS Statistics for Windows, Version 29.0. Armonk, NY: IBM Corp.).

## Results

**Fig. 1:**
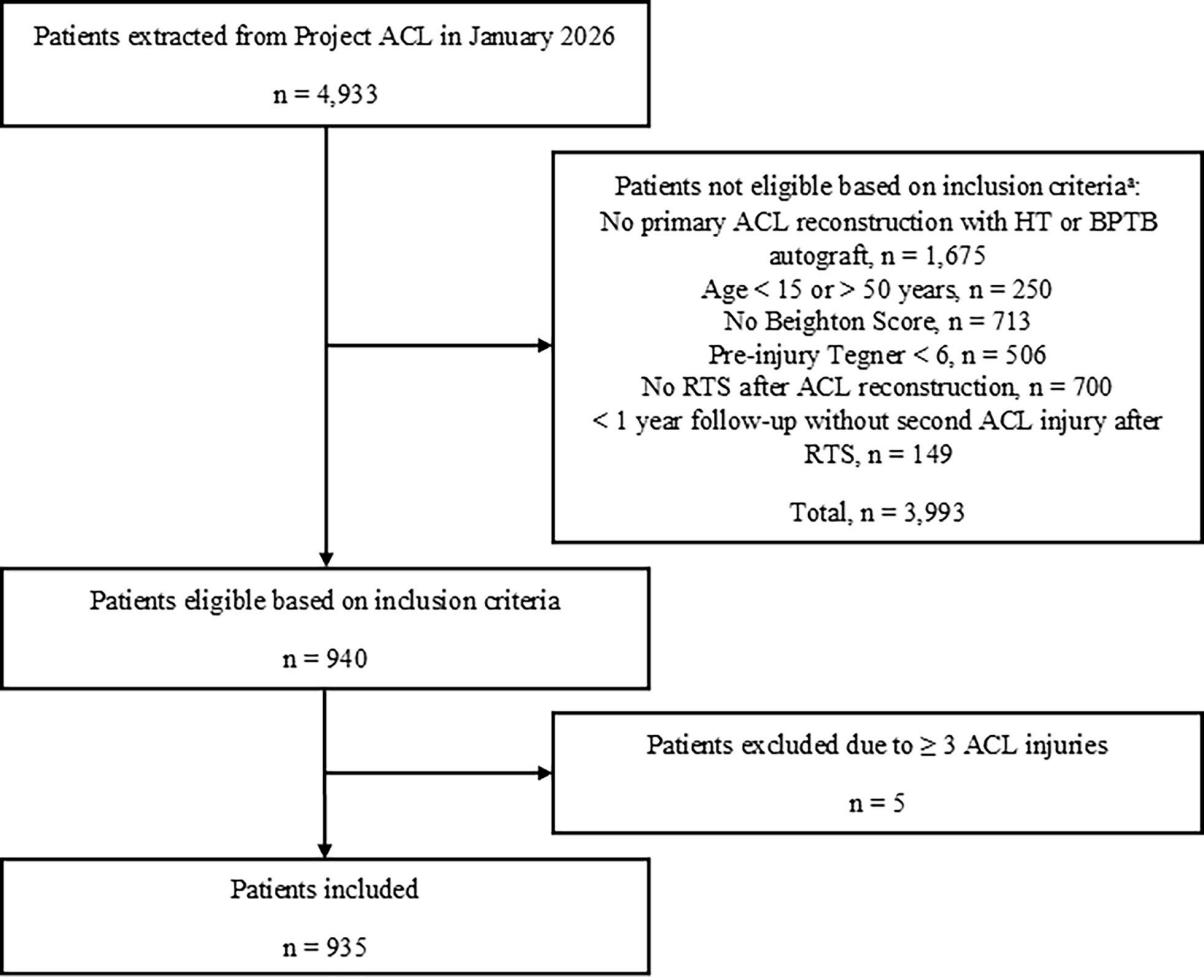
Flowchart representing patient selection, ACL: Anterior cruciate ligament; BPTB: Bone–patellar tendon–bone; HT: Hamstring tendon; *n*: Number of patients, RTS: Return to sport; Tegner: Tegner Activity Scale. ^a^patients may fulfil multiple criteria

Data for a total of 4,933 patients were extracted from Project ACL’s database, of which 935 patients were included (Fig. 1). Of the included patients, 481 (51.4%) were female and the mean age at the time of ACL reconstruction was 23.7 ± 7.8 years (Table 1). The median follow-up time from the time of RTS to the end point was 54.4 months (IQR: 47.5 months), which was significantly different between patients with different total Beighton Scores (*p* < 0.001). Patients with a total Beighton Score of 9 had the shortest median follow-up time after RTS of 24.7 months (IQR: 29.7 months).

**Table 1:** Demographic data for included patients

Patient demographics	Total, *n* = 935
Patient sex, females, *n* (%)	481 (51.4)
Age, years mean ± SD [min–max]	23.7 ± 7.8 [15.0–50.0]
Height, cm mean ± SD	174.8 ± 9.1
Weight, kg mean ± SD	71.9 ± 11.7
BMI, mean ± SD	23.4 ± 2.6
Knee hyperextension, *n* (%)	283 (30.3)
Time from injury to ACL reconstruction, months median (IQR)	3.6 (4.0)
Time from ACL reconstruction to RTS, months median (IQR)	11.8 (4.3)
Follow-up time after RTS, months median (IQR) [min–max]	54.4 (47.5) [0.1–138.6]
Level of sport pre-injury, Tegner median (IQR)	9.0 (1.0)
Level of sport at RTS**,** Tegner median (IQR)	7.0 (2.0)
Beighton Score, median (IQR)	2 (4)
Distribution of patients across each Beighton Score, *n* (%)	
0	370 (39.6)
1	78 (8.3)
2	147 (15.7)
3	65 (7.0)
4	101 (10.8)
5	57 (6.1)
6	47 (5.0)
7	38 (4.1)
8	18 (1.9)
9	14 (1.5)
Graft choice, *n* (%)	
Hamstring tendon autograft	732 (78.3)
Bone–Patellar Tendon–Bone autograft	203 (21.7)
Second ACL injury over the study period, *n* (%)	122 (13.1)
Graft rupture, *n* (%)	69 (56.6)
Contralateral ACL injury, *n* (%)	53 (43.4)
Time from RTS to second ACL injury, months median (IQR)	12.4 (25.1)
Time from RTS to graft rupture, months median (IQR)	7.6 (12.2)
Time from RTS to contralateral ACL injury, months median (IQR)	28.0 (36.6)

BMI: Body mass index; cm: Centimeters; GJH: Generalized joint hypermobility; IQR: Interquartile range; kg: Kilogram; min: Minimum; max: Maximum; *n*: Number of patients; RTS: Return to Sport; SD: Standard deviation; Tegner: Tegner activity scale

**Fig. 2:**
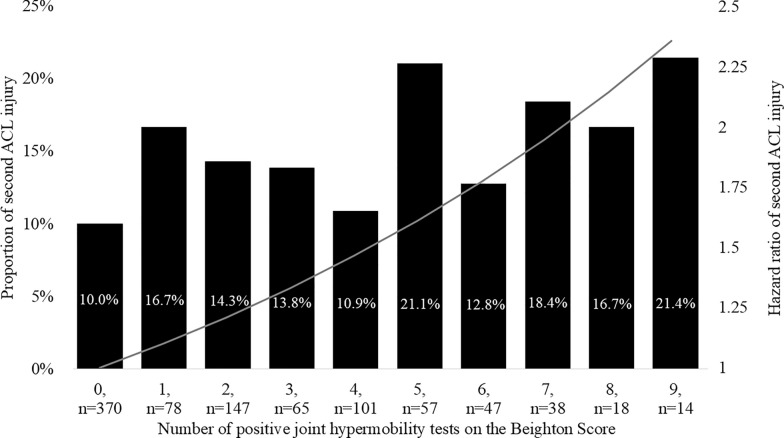
Proportion (black bars), and cumulative hazard ratio (gray line), of second ACL injuries, i.e., graft rupture or contralateral ACL injury, across patients based on the numbers of positive joint hypermobility tests on the Beighton Score at the longest available time after return to sport. ACL: Anterior cruciate ligament; *n*: Number of patients

**Fig. 3:**
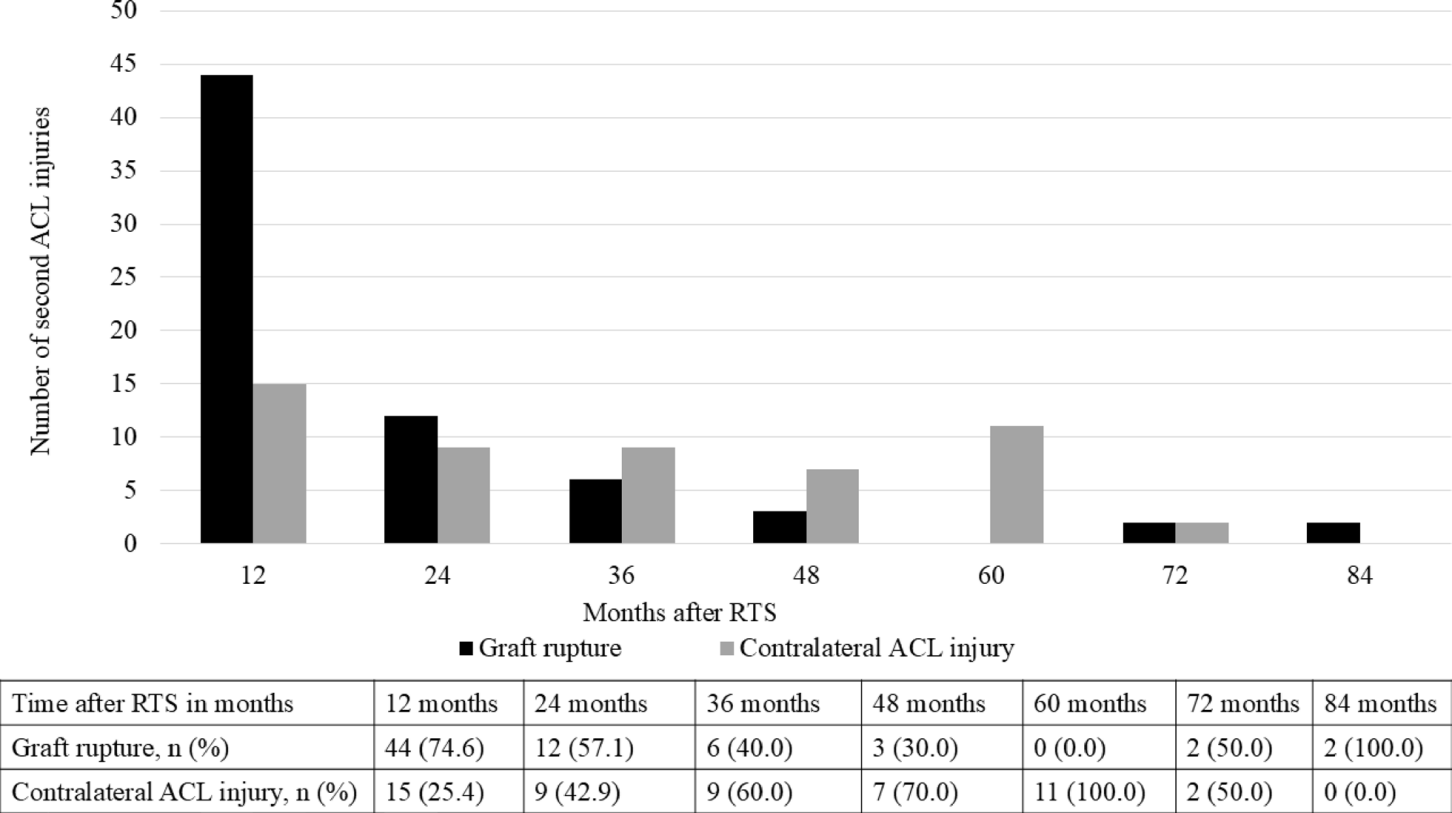
Distribution of second ACL injuries, graft ruptures (black bars) or contralateral ACL injuries (gray bars), for each year after RTS until date of data extraction. ACL: Anterior cruciate ligament; *n*: Number of patients; RTS: Return to sport

Over the study period, the cumulative incidence of second ACL injury was 13.1% (*n* = 122). Specifically, the cumulative incidence of graft rupture was 7.4% (*n* = 69), and 5.7% (*n* = 53) for contralateral ACL injury. The median Beighton Score for all included patients was 2 (IQR: 4). The highest proportion of second ACL injuries were observed in patients with Beighton Scores 9 (21.4%), 5 (21.1%), and 7 (18.4%) (Fig. 2). Figure 3 displays the distribution of second ACL injuries, stratified by graft ruptures or contralateral ACL injuries, for each year after RTS until the study end point.

## Primary Outcome

**Fig. 4:**
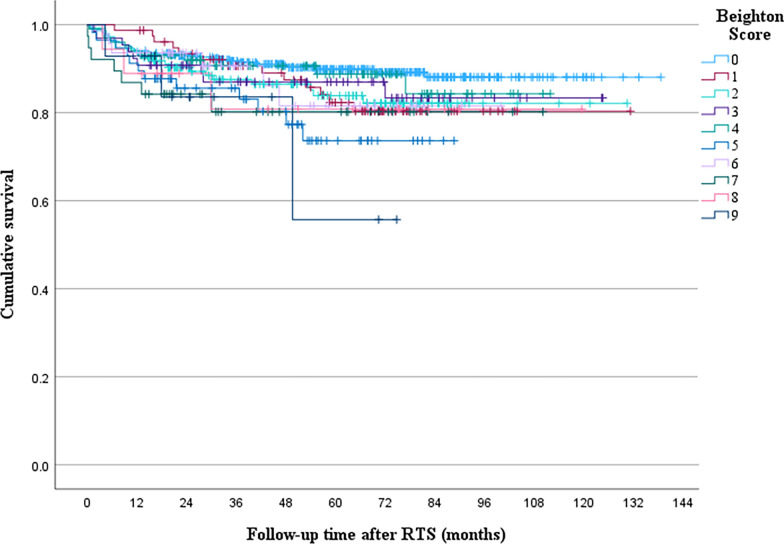
Kaplan–Meier curve on remaining free from second ACL injury after RTS following primary ACL reconstruction, stratified by total Beighton Score. ACL = Anterior cruciate ligament, RTS = Return to sport

Over the study period of median 54.4 months after RTS, the HR of second ACL injury was 1.10 times greater for each positive joint hypermobility test on the Beighton Score (95% CI 1.003–1.22, *p* = 0.044) adjusted for age at primary ACL reconstruction, return to pre-injury level Tegner or higher, choice of autograft, presence of knee hyperextension, and patient sex (Table 2 and Fig. 4). The model demonstrated a possibly helpful discrimination (Harrell’s *C* = 0.621), the proportional hazards assumption was not violated (Grambsch and Therneau’s test *p* = 0.473), the model showed good fit (χ^2^ = 24.4, *p* < 0.001), and the log-linearity assumption was not violated (*p* = 0.761). Data availability throughout the study period is presented for respective 12-month period in Appendix Table 1. Full model estimates for all covariates are presented in Appendix Table 2.

**Table 2:** Unadjusted and adjusted Cox regression model to estimate the hazard ratio of second ACL injury over the study period after return to sport dependent on Beighton Score

	Unadjusted		Adjusted	
Covariate	Hazard ratio (95% CI)	*p* value	Hazard ratio (95% CI)	*p* value
Beighton Score	1.10 (1.03–1.18)	0.006	1.10 (1.003–1.22)	0.044

Adjusted for age at primary ACL reconstruction, return to pre-injury level of Tegner or higher, choice of autograft for ACL reconstruction, presence of knee hyperextension, and patient sex

ACL: Anterior cruciate ligament; CI: Confidence interval; Tegner: Tegner activity scale

Number of second ACL injuries, i.e., graft rupture or contralateral ACL injury, *n* = 122

## Secondary Outcomes

**Fig. 5:**
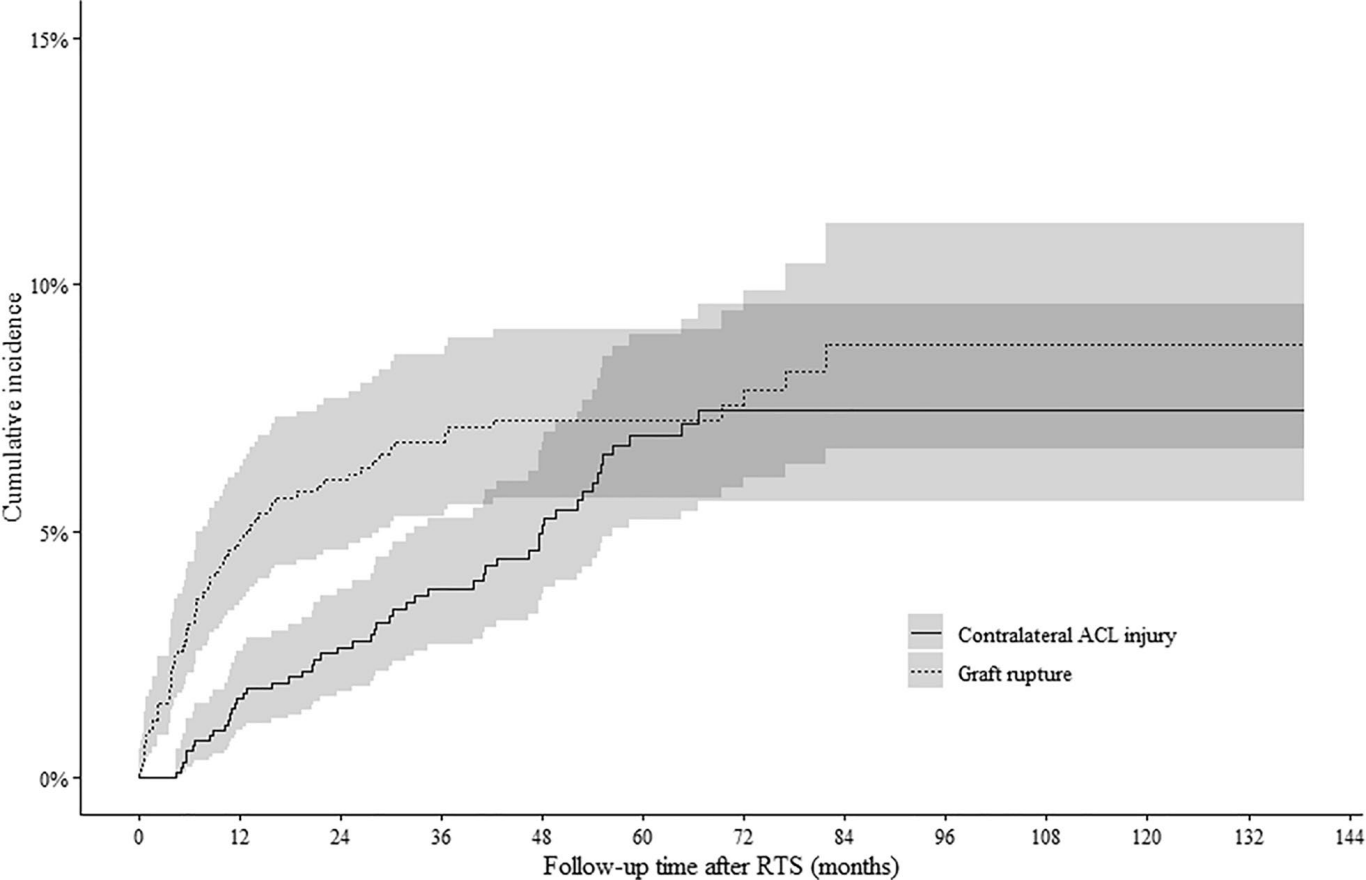
Cumulative incidence functions on graft rupture and contralateral ACL injury after RTS following primary ACL reconstruction. ACL = Anterior cruciate ligament, RTS = Return to sport

Over the study period, the adjusted HR of graft rupture was 1.15 times greater for each positive joint hypermobility test on the Beighton Score (95% CI 1.01–1.30, *p* = 0.031) (Table 3 and Fig. 5). For the graft rupture analysis, the model demonstrated a possibly helpful discrimination (Harrell’s *C* = 0.660), the proportional hazards assumption was not violated (Grambsch and Therneau’s test *p* = 0.466), the model showed good fit (χ^2^ = 22.9, *p* = 0.001), and the log-linearity assumption was not violated (*p* = 0.270). For contralateral ACL injury, there was no difference in the adjusted HR based on the number of positive joint hypermobility tests on the Beighton Score (HR: 1.04, 95% CI 0.89–1.21, *p* = 0.598). For the contralateral ACL injury analysis, the model demonstrated a poor discrimination (Harrell’s *C* = 0.599), the proportional hazards assumption was not violated (Grambsch and Therneau’s test *p* = 0.962), the model did not show good fit (χ^2^ = 8.22, *p* = 0.222), and the log-linearity assumption was not violated (*p* = 0.376). Full model estimates for all covariates are presented in Appendix Table 2.

**Table 3:** Unadjusted and adjusted Cox regression model to estimate the hazard ratio of graft rupture and contralateral ACL injury over the study period after return to sport dependent on Beighton Score

	Unadjusted		Adjusted	
Outcome/covariate	Hazard ratio (95% CI)	*p* value	Hazard ratio (95% CI)	*p* value
Graft rupture				
Beighton Score	1.10 (1.002–1.20)	0.044	1.15 (1.01–1.30)	0.031
Contralateral ACL injury				
Beighton Score	1.10 (0.99–1.23)	0.065	1.04 (0.89–1.22)	0.598

Adjusted age at primary ACL reconstruction, for return to pre-injury level of Tegner or higher, choice of autograft for ACL reconstruction, presence of knee hyperextension, and patient sex

ACL: Anterior cruciate ligament; CI: Confidence interval; Tegner: Tegner activity scale

Number of graft ruptures: *n* = 69; number of contralateral ACL injuries: *n *= 53

## Discussion

The main finding of this study was that for each unit increase in the number of positive joint hypermobility tests on the Beighton Score, the HR of second ACL injury after RTS increased by 10%, adjusted for age at primary ACL reconstruction, return to pre-injury Tegner level or higher at RTS, choice of autograft for primary ACL reconstruction, presence of knee hyperextension, and patient sex. The association between Beighton Score and the composite second ACL injury endpoint appeared primarily attributable to graft rupture (HR: 1.15, 95% CI 1.01–1.30, *p* = 0.031), whereas the estimate for contralateral ACL injury was smaller and inconclusive (HR: 1.04, 95% CI 0.89–1.22, *p* = 0.598). These findings suggest that clinicians should consider the number of positive joint hypermobility tests, rather than solely rely on a binary GJH classification when assessing and counseling patients on the risk of a second ACL injury after ACL reconstruction.

Patients with GJH have previously been reported to be at greater risk for a second ACL injury compared with patients without GJH [5, 26, 32]. A previous study reported 4.24 times greater HR of second ACL injury in patients with GJH compared to patients without GJH [5]. However, the use of different Beighton Score cutoffs for dichotomizing GJH may result in misclassification, which can lead to either overestimate or underestimate the presence of GJH. The greater HR observed with increasing numbers of positive joint hypermobility tests on the Beighton Score, suggests that GJH exists on a spectrum rather than being dichotomous characterized by a clear threshold. Consequently, our findings of 10% exponential increase in second ACL injury HR after RTS may underscore the importance of more refined assessment of joint hypermobility. Specifically, our findings suggest that the number of positive joint hypermobility tests, rather than the mere presence of GJH, should be considered in individualized rehabilitation and risk stratification after primary ACL reconstruction.

The use of the traditional binary classification of GJH based on the Beighton Score, Larson et al. [26], and Zsidai et al. [5] reported that patients with GJH had a second ACL injury occurrence of 34.1% (mean follow-up time: 72 months; age range 12–62 years), and 38.2% (≤ 84 month follow-up time; age range: 16–50 years), respectively, in contrast to 7.7%, and 10.1%, respectively, in patients without GJH. In the present study, the overall occurrence of second ACL injury over a median follow-up time of 48 months was 12.9%, where no classification of GJH was made. Nonetheless, previous studies [5, 26] have used two different cutoff points for the classification of the GJH phenotype. According to the most recent consensus [8], different cutoff points for GJH are advocated with increased threshold for females and younger individuals, since joint hypermobility is more common in females and in the younger population. The need for different cutoff points for GJH in different sub-populations is further highlighted in a study by Singh et al. [29] which reported that only a minority of patients were correctly classified with GJH when a single cutoff point was utilized on the Beighton Score. Nevertheless, the risk of misclassification of GJH is still possible even if different cutoff points in different sub-populations are applied.

The results of this study offer an alternative to binary classification of GJH, since we show that the HR of second ACL injury increases exponentially with each positive joint hypermobility test on the Beighton Score. For example, based on the adjusted HR of 1.10 per point, patients with 1, 2, or 3 positive Beighton Score tests had an estimated 10%, 21%, or 33% higher HR of second ACL injury, respectively, compared to patients with a score of 0. Consequently, although GJH assessment using a standardized method, such as the Beighton Score, remains valuable for second ACL injury risk stratification, interpretation should not only consider the possible presence of GJH, but also the degree and extent of joint hypermobility. Patients should be counseled and informed accordingly with regard to the potential increased HR of second ACL injury after RTS after primary ACL reconstruction. Future research should aim to identify rehabilitation and surgical factors that increase the likelihood of a RTS with minimal risk of re-injury for high-risk patients, and, if none are sufficient, explore the option of advising against RTS.

The timing of second ACL injuries differed substantially between components: graft ruptures occurred earlier after RTS (median 7.6 months), whereas contralateral ACL injuries occurred later (median 28 months). This observed temporal separation was consistent with previous literature on graft rupture and contralateral injury [33], which reflects partly distinct processes and may partly explain why the composite second ACL injury association was more apparent for graft rupture than for contralateral ACL injury. For clinicians, the results suggest to prioritize early post-RTS strategies aimed at reducing graft failure risk, while recognizing that contralateral injury risk may emerge later and warrants continued preventive efforts beyond the first year.

### Limitations

The presented study has some limitations. First, the degree of hypermobility in the joint, i.e., joint angle was not accounted for, but only the threshold for a positive test in the Beighton Score was considered. Furthermore, individual tests scores on the Beighton Score could not be accounted for. Second, only the reported level of physical activity on the Tegner was analyzed in this study, and not the exact sport or exposure time the patients were undertaking in their sport. Greater and more intense exposure to knee-demanding maneuvers would likely increase the risk for second ACL injury. Third, only 117 (12.5%) patients included in the study had Beighton Score ≥ 6, which may potentially limit the statistical precision of our analysis. Fourth, the results of this study could be affected by potential concomitant ligamentous or intra-articular injuries, which include medial collateral ligament injury [34] or lateral meniscus injury [35], which are not reported in Project ACL. Fifth, we could not account for additional surgical procedures, such as the lateral extra-articular tenodesis, performed in addition to the ACL reconstruction, which has been shown to reduce the risk of second ACL injury [36]. Finally, the use of complete case analysis assumes that data are missing completely at random, which cannot be verified and may introduce bias if this assumption is violated.

## Conclusion

The HR of second ACL injury (graft rupture or contralateral ACL injury) after RTS in patients with primary ACL reconstruction increased by 10%, and by 15% for isolated graft rupture, for each positive joint hypermobility test on the Beighton Score, while no association was observed for contralateral ACL injury. The presented findings should prompt clinicians to consider the number of positive joint hypermobility tests, and not solely rely on binary GJH classification, when assessing risk for second ACL injury after ACL reconstruction.

## Data Availability

The data that support the findings of this study are available on reasonable request from the corresponding author. The data are not publicly available due to privacy or ethical restrictions.

## Appendix

See Tables 1, 2.

**Table 1:** Patients at risk for second ACL injury across different timepoints after RTS

Point in time after RTS, months	At RTS	12	24	36	48	60	72	84	96	108	120	132	144
Number of positive hypermobile joints on the Beighton Score, n													
0	370	348	321	280	235	182	130	72	37	20	8	2	0
1	78	77	70	60	55	44	25	11	6	1	1	0	
2	147	138	104	86	73	59	40	18	4	3	2	0	
3	65	60	51	45	38	32	23	12	5	2	2	0	
4	101	95	80	62	55	43	27	13	7	2	0		
5	57	52	38	34	28	13	6	2	0				
6	47	44	32	22	18	13	8	2	1	0			
7	38	33	23	17	13	13	8	3	2	1	0		
8	18	16	11	10	9	8	7	4	1	1	0		
9	14	13	8	4	3	2	1	0					

ACL: Anterior cruciate ligament; *n*: Number of patients; RTS: Return to sport

**Table 2:** Full estimates of the adjusted Cox regression model to estimate the hazard ratio of second ACL injury over the study period after return to sport dependent on Beighton Score

	Second ACL injury (graft rupture or contralateral ACL injury)		Graft rupture		Contralateral ACL injury	
Covariate	Hazard ratio (95% CI)	*p* value	Hazard ratio (95% CI)	*p* value	Hazard ratio (95% CI)	*p* value
Beighton Score	1.10 (1.003–1.22)	0.044	1.15 (1.01–1.30)	0.031	1.04 (0.89–1.22)	0.598
Age at primary ACL reconstruction	0.95 (0.92–0.98)	< 0.001	0.93 (0.90–0.98)	0.002	0.96 (0.92–1.01)	0.086
Return to pre–injury level of Tegner or higher	0.89 (0.62–1.28)	0.533	0.82 (0.50–1.33)	0.415	0.99 (0.57–1.71)	0.970
Autograft (HT vs BPTB)	0.72 (0.45–1.14)	0.164	0.50 (0.25–1.003)	0.051	1.05 (0.56–1.98)	0.878
Knee hyperextension	0.84 (0.50–1.42)	0.518	0.64 (0.32–1.27)	0.636	1.25 (0.57–2.75)	0.584
Patient sex (female vs male)	1.09 (0.75–1.58)	0.663	1.37 (0.84–2.25)	0.210	0.79 (0.44–1.41)	0.426

Adjusted for age at primary ACL reconstruction, return to pre-injury level of Tegner or higher, choice of autograft for ACL reconstruction, presence of knee hyperextension, and patient sex

Number of second ACL injuries, i.e., graft rupture or contralateral ACL injury, *n* = 122. Number of graft ruptures, *n* = 69. Number of contralateral ACL injuries, *n* = 53

ACL: Anterior cruciate ligament; BPTB: Bone–patellar tendon–bone; CI: Confidence interval; HT: Hamstring tendon; Tegner: Tegner activity scale

## Abbreviations

ACL: Anterior cruciate ligament
BMI: Body mass index
CI: Confidence interval
Cm: Centimeters
GJH: Generalized joint hypermobility
HR: Hazard ratio
HT: Hamstring tendon
IQR: Interquartile range
Kg: Kilograms
N: Number
RTS: Return to sport
SD: Standard deviation
Tegner: Tegner activity scale
